# Schizophrenics can be good mothers too

**DOI:** 10.1192/pb.bp.115.051011

**Published:** 2017-02

**Authors:** Duncan Double

To protect her children the author of this book chose to publish it under a pseudonym, Q. S. Lam. However, it's easy to break her anonymity and she accepts it can only be partial. She is a British Bangladeshi artist who has had several psychotic episodes, including postpartum. She has been diagnosed with schizoaffective disorder but prefers to describe herself as having a different sort of brain.

Her friend Stephen Fry has described the book as ‘brilliant’ – an endorsement displayed on the book cover – and Alastair Campbell has tweeted the same. Artwork and poetry complement the narrative of the author's personal and family history, which includes episodes of psychosis, and the description of the dissociated parts of herself and how she recovers. She does not take antipsychotic medication.

She makes remarks – not always very complimentary – about each mental health practitioner that she has seen over the years, dating back to the time when she first sought help. Also discussed is the impact of her mental health problems on her husband and children. She moved to Brussels, as her husband works there.

The psychiatrist she has most identified with is Erik Thys, who is also an artist. He did not advise Q.S. not to have a second child; instead, he said it was ‘doable’. Q.S. openly questions whether it was fair on her children that she became a mother and dedicates the book to them.

The strength of this book is its honesty. Q.S. has experienced multiple sexual assaults by men and considers whether her mental health issues are a sane response to an insane situation. She reveals her heart and mind, truthfully expressing what she feels and thinks, and I found the directness of the book attractive. As Q.S. notes, doctors generally don't like their authority to be challenged. However, in my opinion there needs to be an open discussion about the stigmatisation of mothers with mental health problems.

